# Transcriptome and Metabolome Analyses Reveal the Mechanism of Color Differences in Pomegranate (*Punica granatum* L.) Red and White Petals

**DOI:** 10.3390/plants14050652

**Published:** 2025-02-20

**Authors:** Yong Zhang, Peng Han, Ruijie Zhao, Shuhan Yu, Hang Liu, Shuren Ji, Wei Chen

**Affiliations:** 1 School of Landscape Architecture and Horticulture, Jiangsu Agri-Animal Husbandry Vocational College, Taizhou 225300, China; 2022010535@jsahvc.edu.cn (Y.Z.); liuhang316022@126.com (H.L.); 2 College of Agro-Grassland Science, Nanjing Agricultural University, Nanjing 210095, China; 19852869215@163.com (P.H.); zrj152342767970520@163.com (R.Z.); 3 College of Landscape Architecture, Zhejiang A&F University, Hangzhou 311300, China; 20220144@zafu.edu.cn; 4 College of Landscape Architecture, Jiangsu Vocational College of Agriculture and Forestry, Jurong 212400, China; jsr19901205@163.com

**Keywords:** anthocyanin, carotenoid, metabolome, *Punica granatum*, petal, RNA-seq

## Abstract

Pomegranate (*Punica granatum* L.) is an important economic tree, possessing both edible and ornamental value. Flower color is an important ornamental trait of pomegranate, but the color formation pattern and related molecular mechanisms of pomegranate petals are still unclear. In this study, we conducted physiological, transcriptomic, and metabolomic studies on the petals of Tunisia and White pomegranate varieties during the blooming stage. The results showed that compared to White petals, the contents of anthocyanin, carotenoid, and sucrose in Tunisia petals were significantly increased, while the flavonoid content was significantly decreased. Through RNA-seq, 23 DEGs were identified in the anthocyanin synthesis, and 3 DEGs were identified in the carotenoid synthesis. Transcription factor genes such as *MYB*, *bHLH*, *WRKY*, and *MADS* were identified as key candidates for regulating anthocyanin metabolism. Metabolomic analysis revealed that eight DEMs are associated with anthocyanin synthesis and three DEMs are associated with carotenoid synthesis. In addition, caffeic acid and its derivatives were significantly upregulated in Tunisia petals. In summary, we propose the following hypothesis: the accumulation of anthocyanins and carotenoids is the reason for the red color of Tunisian petals, and the upregulation of structural genes, including *PAL*, *C4H*, *4CL*, *CHS*, *CHI*, *F3H*, *F3′H*, *DFR*, *ANS*, *PSY*, and *LCYB*, leads to an increase in their content. Transcription factor genes such as *MYB*, *bHLH*, *bZIP*, *MADS*, and *WRKY* may also play a positive role in anthocyanin accumulation. The research results provide a basis for the theory of pomegranate petal color formation.

## 1. Introduction

Pomegranate (*Punica granatum* L.) is a deciduous tree or shrub belonging to the genus Punica in the Lythraceae family, and it is an ancient crop originating in the Himalayas from Iran to northern India [1]. Pomegranates are cultivated in both temperate and tropical regions worldwide, and there are many varieties, which can be divided into fruit varieties and ornamental varieties according to their purposes [2]. The color of pomegranate flower petals varies depending on the variety, mainly including red and white, which is one of the most important ornamental traits of pomegranate [3]. The formation mechanism of flower petal coloration is intricate, resulting from the synergistic effects of multiple factors, with the composition and content of pigments playing a decisive role [4]. The pigments that determine the color of flower petals include flavonoids/anthocyanins, carotenoids, and betalains, among which flavonoids/anthocyanins and carotenoids are the basis for the formation of the color of most plant flower petals [5,6].

Anthocyanins are water-soluble pigments belonging to the flavonoid class of compounds [7]. There are various types of anthocyanins in plants, the six most common of which are pelargonidin (Pg), cyanidin (Cy), delphinidin (Dp), peonidin (Pn), malvidin (Mv), and petunidin (Pt) [8]. The genes that control anthocyanin synthesis are divided into structural genes and regulatory genes, among which structural genes encode enzymes involved in anthocyanin synthesis, including *phenylalanine ammonia-lyase* (*PAL*), *cinnamate 4-hydroxylase* (*C4H*), *4-coumarate-CoA ligase* (*4CL*), *chalcone synthase* (*CHS*), *chalcone isomerase* (*CHI*), *flavanone3-hydroxylase* (*F3H*), *flavonoid 3′-hydroxylase* (F*3′H*), *flavonoid3′5′-hydroxylase* (*F3′5′H*), *flavonol synthase* (*FLS*), *dihydroflavonol 4-reductase* (*DFR*), *anthocyanidin synthase* (*ANS*), and *UDPG-flavonoid-3-O-glycosyltranferase* (*UFGT*) [9]. In the process of anthocyanin synthesis, transcription factors (TFs) promote or inhibit the biosynthesis of anthocyanins in plants by regulating the expression levels of structural genes. Transcription factor families such as *MYB*, *bHLH*, *WD40*, and *bZIP* can all participate in the regulation of anthocyanin biosynthesis [10,11].

Carotenoids are a class of fat-soluble compounds, which can be divided into two major categories: carotenes and xanthophylls [12]. The biosynthesis of carotenoids begins with geranylgeranyl diphosphate (GGPP), which is synthesized through the methylerythritol 4-phosphate (MEP) pathway [13]. GGPP ultimately forms various types of carotenoids, which are catalyzed by a series of enzymes, including phytoene synthase (PSY), Phytoene desaturase (PDS), ζ-Carotene isomerase (Z-ISO), Carotene isomerase (CRTISO), ζ-Carotene desaturase (ZDS), Lycopene β-cyclase (LCYB), Lycopene ε-cyclase (LCYE), β-Carotene hydrolase (BCH), Cytochrome P450 carotene β-hydroxylase (CYP97A), Cytochrome P450 carotene ε-hydroxylase (CYP97C), Zeaxanthin epoxidase (ZEP), violaxanthin de-epoxidase (VDE), and Neoxanthin synthase (NXS) [14,15]. Transcription factors also play a significant role in carotenoid biosynthesis. Transcription factor genes that regulate plant carotenoid synthesis include *MYB*, *bHLH*, *MADS*, *bZIP*, *AP2*/*ERF*, and *WRKY* [16,17,18].

Currently, research related to pomegranate pigment primarily focuses on the mechanisms of fruit coloring and peel coloring, while there are relatively few studies on the color differences among pomegranate petals [19,20,21,22]. To comprehensively analyze the formation mechanism of pomegranate petal color, this study utilized petals from Tunisia and White pomegranate varieties as materials for transcriptome, metabolome, and physiological level research, aiming to identify key structural genes and transcription factors related to petal color formation. This study will provide new insights into the mechanism of color formation in pomegranate petals.

## 2. Results

### 2.1. Pomegranate Petal Color Phenotype

**Figure 1:**
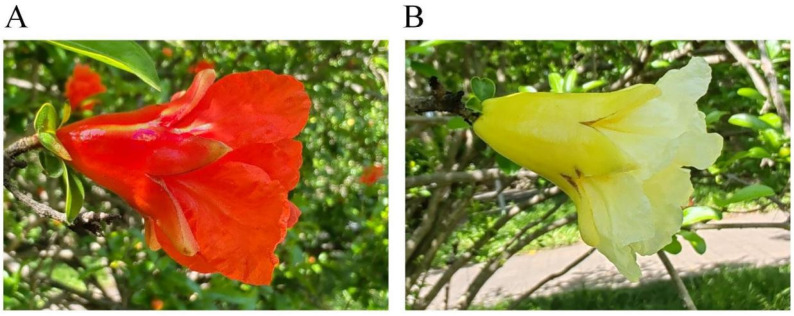
The flowers of pomegranate varieties for testing: (**A**) “Tunisia” pomegranate cultivars; (**B**) “White” pomegranate cultivars.

The petals of Tunisia and White are red and white, respectively (Figure 1). The L* value represents the brightness of the color, and the higher the L* value, the closer it is to white. The a* value represents redness, with a positive value indicating a reddish hue and a negative value indicating a greenish hue. The b* value represents yellowing; a positive value indicates a yellowish color, while a negative value indicates a bluish color. The C* value represents color saturation, and the higher the C* value, the brighter the color. As shown in Table 1, the phenotypic parameters of Tunisia and White pomegranate are consistent with their color phenotypes.

### 2.2. Physiological Indicators of Pomegranate Petal

**Figure 2:**
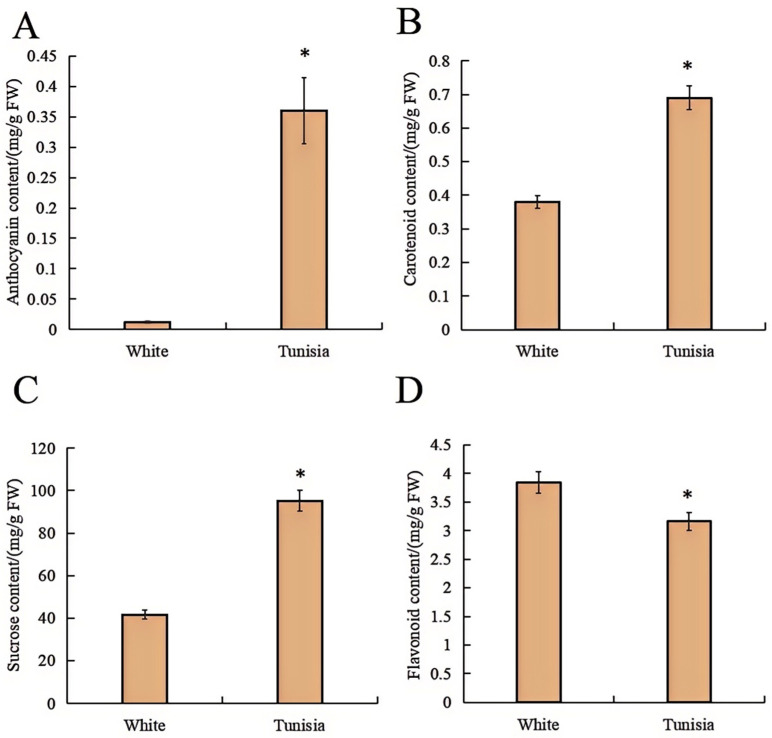
Comparison of physiological indicators between Tunisia and White petals: (**A**), Anthocyanin content; (**B**), Carotenoid content; (**C**), Sucrose content; (**D**), Flavonoid content. * indicates significant differences at *p* < 0.05 level.

The relative contents of anthocyanin, flavonoid, carotenoid, and sucrose in the petals of Tunisia and White were determined. The results showed that the anthocyanin content, carotenoid content, and sucrose content of Tunisia petals were significantly higher than those of White petals, which were 0.36 mg/g, 0.69 mg/g, and 95.26 mg/g, respectively (Figure 2). However, the carotenoid content of Tunisia petals was significantly lower than that of White petals, which was 3.16 mg/g (Figure 2).

### 2.3. RNA-Seq Data Overview

Raw data (accession number: PRJNA1224964)were obtained by sequencing six pomegranate petal samples, and clean reads were further obtained after filtering the raw reads (Table S1). The clean base of each sample was higher than 8 Gb, and the overall sequencing error rate was less than 0.01% (Table S1). The Q20 values of all six sequencing samples were greater than 98%, and the Q30 values were all greater than 96% (Table S1). In addition, in this sequencing, the GC content was higher than 50% in all six samples (Table S1). The percentage of reads aligned to the reference genome (reads mapped) was greater than 97%, the percentage of unique reads aligned to the reference genome (uniquely mapped) was greater than 93%, and the percentage of multiple reads aligned to the reference genome (multi-mapped) ranged from 3.52% to 4.55% (Table S2). The absolute values of the Pearson correlation coefficients (r) for the three biological replicates of Tunisia and White samples were greater than 0.98 and 0.97, respectively, indicating good reproducibility for both samples (Figure S1).

### 2.4. DEG Identification and KEGG Enrichment Analyses

**Figure 3:**
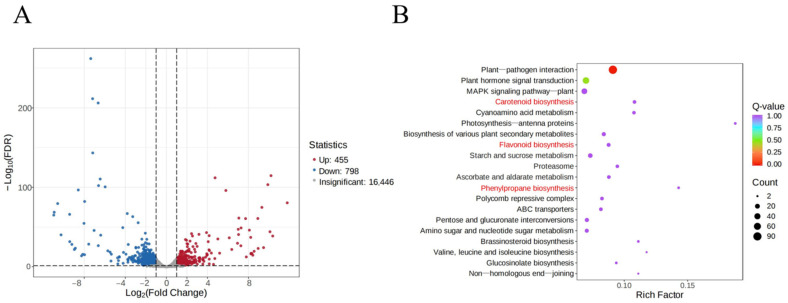
Differentially expressed gene (DEG) identification and KEGG analysis: (**A**) volcano plot of DEGs from Tunisia vs. White; (**B**) top 20 metabolic pathways analyzed with KEGG enrichment for DEGs from Tunisia vs. White. The pathways associated with pigment metabolism are highlighted in red.

A total of 17,699 genes were obtained from RNA-seq of pomegranate petals. Based on the screening criteria for DEGs (|log_2_Fold Change| ≥ 1 and FDR < 0.05), a total of 1253 DEGs were identified in the Tunisia vs. White comparison, of which 455 (36.31%) were upregulated and 798 (63.69%) were downregulated (Figure 3A). KEGG enrichment analysis revealed that these DEGs were significantly enriched in 13 metabolic pathways, among which the pathways related to flower color included flavonoid, carotenoid, and phenylpropanoid biosynthesis (Figure 3B).

### 2.5. Analysis of Key Genes Associated with Pigment

A total of 26 DEGs related to pigment were identified between Tunisia petals and White petals, which are involved in three metabolic pathways: phenylpropane biosynthesis, flavonoid biosynthesis, and carotenoid biosynthesis (Table 2). In the phenylpropanoid biosynthesis pathway, two *PAL*, one *C4H*, and two *4CL* exhibited upregulated expression (Table 2). Eighteen DEGs were identified in the flavonoid biosynthesis pathway, namely, two *CHS* (upregulated expression), four *CHI* (three upregulated and one downregulated), one *F3H* (upregulated expression), five *F3′H* (four upregulated and one downregulated), three *DFR* (two upregulated and one downregulated), and three *ANS* (two upregulated and one downregulated) (Table 2). In the carotenoid biosynthesis pathway, two *PSY* and one *LCYB* exhibited upregulated expression (Table 2).

### 2.6. Screening Pigment Metabolism-Related TFs

**Figure 4:**
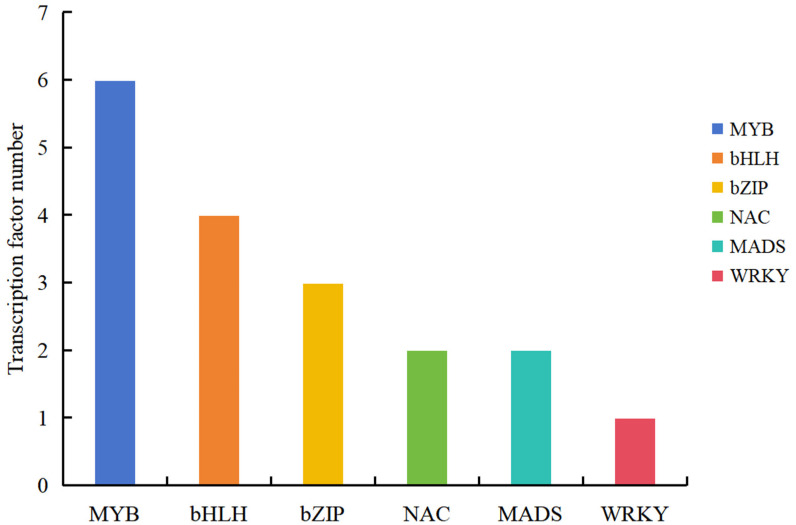
Statistics of differentially expressed Transcription factors.

Using PlantTFDB prediction, 18 DEGs encoding transcription factors (TFs) were identified (Figure 4): *MYB* (5 upregulated and 1 downregulated), *bHLH* (4 upregulated), *bZIP* (3 upregulated), *NAC* (2 upregulated), *MADS* (2 upregulated), and *WRKY* (1 upregulated) (Table S3). This result indicates that transcription factor genes are also involved in the regulation of pigment synthesis in pomegranate petals.

### 2.7. Metabolome Data Overview

The TIC (total ion chromatogram) plot of the mass spectrometry detection of QC (quality control) samples reveals high overlap in the metabolite detection curves, with consistent retention times and peak intensities (Figure S2). This indicates good stability of the mass spectrometry signal for the same sample at different time points. According to the analysis of the empirical cumulative distribution function, substances with a CV (coefficient of variation) value of less than 0.5 account for over 85% of the QC samples, and substances with a CV value of less than 0.3 account for over 75%, indicating that the experimental data are stable and reliable (Figure S3). Based on the PCA results of the samples (Figure S4), it can be observed that there is a significant difference in metabolite distribution between Tunisia petals and White petals, and the reproducibility of the two samples is good.

### 2.8. Orthogonal Partial Least Squares Discriminant Analysis (OPLS-DA)

**Figure 5:**
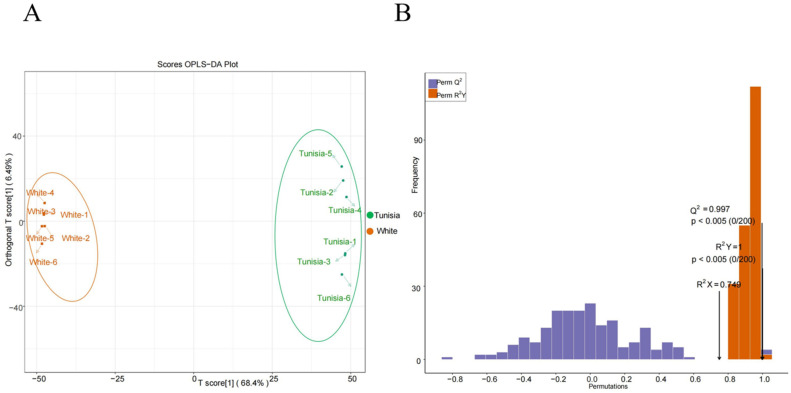
OPLS-DA and model validation: (**A**), OPLS-DA score plot; the horizontal axis represents the predicted principal components, the vertical axis represents the orthogonal principal components, and the percentage represents the explanatory power of the component on the dataset; (**B**), the horizontal axis represents the R^2^Y and Q^2^ values of the model, and the vertical axis represents the frequency of the model’s classification performance in 200 random permutation and combination experiments. The orange represents the random grouping model R^2^Y, the purple represents the random grouping model Q^2^, and the black arrows represent the R^2^X, R^2^Y, and Q^2^ values of the original model.

The obtained mass spectrum data were analyzed using OPLS-DA. It can be seen that there is a large difference between the two sets of mass spectrometry data, while the difference in intragroup repetition between the two sets of mass spectrometry data is small (Figure 5A). The OPLS-DA model was validated, and the predictive parameters of the OPLS-DA evaluation model included Q^2^ = 0.997, R^2^X = 0.749, and R^2^Y = 1 (Figure 5B). The Q^2^ value of the OPLS-DA model was greater than 0.9, indicating that the evaluation model was reliable and effective.

### 2.9. DEM Identification and KEGG Enrichment Analyses

**Figure 6:**
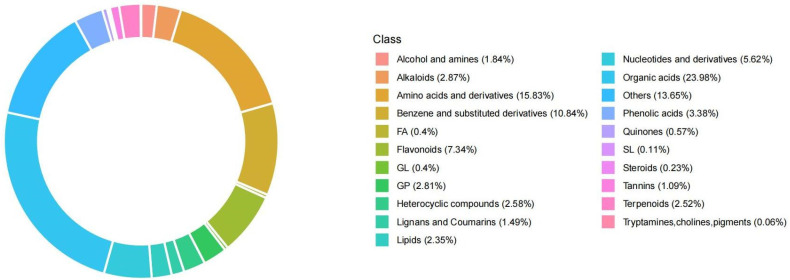
Statistics of metabolite category composition. Each color represents a category of metabolites, and the area of the color block indicates the proportion of that category.

A total of 2549 metabolites were obtained from the metabolomic analysis of pomegranate petals, which were divided into 21 categories (Figure 6). Among them, flavonoids accounted for 7.34%, while trypsin, choline, and pigments accounted for 0.06%, which may be related to pigment in pomegranate petals (Figure 6).

**Figure 7:**
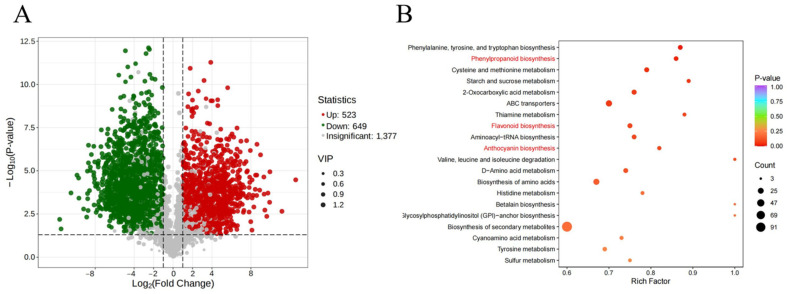
DEM identification and KEGG analysis: (**A**) volcano plot of DEMs from Tunisia vs. White; (**B**) top 20 metabolic pathways analyzed with KEGG enrichment for DEMs from Tunisia vs. White. The pathways associated with pigment metabolism are highlighted in red.

To investigate the metabolic differences between Tunisia petals and White petals, we screened for differentially expressed metabolites (DEMs) between the two using the criteria of VIP > 1 and |Log2FC| ≥ 1.0. A total of 1172 DEMs were identified, of which 523 (44.62%) were upregulated and 649 (55.38%) were downregulated (Figure 7A). To investigate the specific functions of these DEMs, they were annotated in the KEGG database. These differential metabolites were significantly enriched in 16 metabolic pathways, among which 3 pathways, namely, phenylpropanoid biosynthesis, flavonoid biosynthesis, and anthocyanin biosynthesis, were associated with petal color (Figure 7B).

### 2.10. Analysis of Key Metabolites Associated with Pigment

A total of 11 DEMs related to pigment were identified between Tunisia petals and White petals, all of which were upregulated in Tunisia petals (Table 3). Among them, there is one differential metabolite in the phenylpropanoid biosynthesis pathway, which is 4-Coumaroyl-CoA; there are four differential metabolites in the flavonoid biosynthesis pathway, namely, dihydrokaempferol, dihydroquercetin, pelargonidin, and cyanidin; and there are three differential metabolites in the anthocyanin biosynthesis pathway, namely, cyanidin 3-O-glucoside, peonidin 3-O-glucoside, and pelargonidin 3-O-glucoside (Table 3). In addition, there are three differential metabolites in the carotenoid biosynthesis pathway, which are prephytoene diphosphate, phytoene, and 7,8-Dihydro-beta-carotene.

### 2.11. Validation of DEGs with qRT-PCR

**Figure 8:**
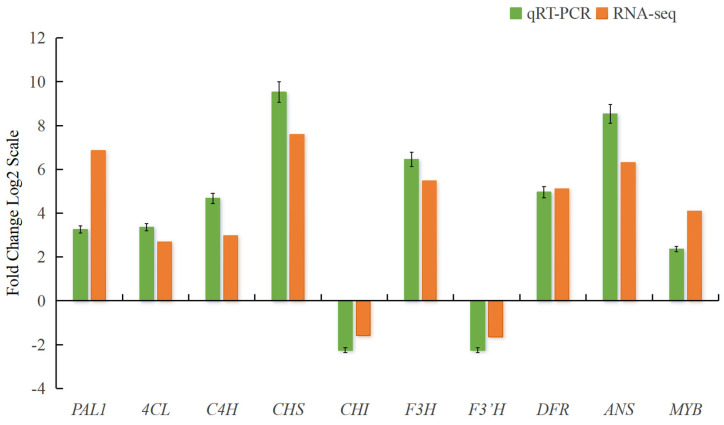
qRT−PCR verification of DEGs. Ordinate shows the logarithm of the differential multiples of the corresponding gene, and the positive and negative values of the *y*-axis express the gene’s upregulated expression and downregulated expression, respectively.

Based on the transcriptome analysis results, we selected 10 DEGs, namely, *PAL1*, *4CL*, *C4H*, *CHS*, *CHI*, *F3H*, *F3′H*, *DFR*, *ANS*, and *MYB*, for qRT-PCR validation. The results indicated that the trend of changes in these 10 DEGs was largely consistent with the transcriptome sequencing results (Figure 8), suggesting that the transcriptome sequencing data of pomegranate petals were accurate and reliable.

## 3. Discussion

### 3.1. Pomegranate Petal Phenotype and Physiological Indicators

Colorimetry is the most effective method for identifying differences in plant color phenotypes. To date, many scholars have identified flower color phenotypes in various ornamental plants such as chrysanthemum (*Chrysanthemum × morifolium*) [23], rose (*Rosa chinensis*) [24], ginger lily (*Hedychium coronarium*) [25], osmanthus (*Osmanthus fragrans*) [26], and peony (*Paeonia suffruticosa*) [27]. This study measured the color phenotype of Tunisia and White pomegranate varieties using a colorimeter (Table 1), and the results showed that there were significant differences in color phenotype between these two petals.

Flower color is an important ornamental trait of ornamental plants, among which anthocyanins and carotenoids are important pigments for color formation [28]. In this study, the anthocyanin and carotenoid content of Tunisia petal was significantly higher than that of White petal (Figure 2A,B), indicating that the difference in anthocyanin and carotenoid content is the main reason for the different flower colors between Tunisia and White varieties (Table 1). The sucrose content of Tunisian petals is significantly higher than that of White petals (Figure 2C), and sucrose content may promote the biosynthesis of plant anthocyanins, which is consistent with previous research results [29]. The flavonoid content of Tunisia petals is significantly lower than that of White petals (Figure 2D). We speculate that there is a competitive relationship between the flavonoid synthesis pathway and the anthocyanin synthesis pathway [30]. More metabolites in Tunisia petals are allocated to anthocyanin synthesis, resulting in a decrease in their flavonoid content.

### 3.2. Key Genes Related to Pomegranate Petal Pigment

**Figure 9:**
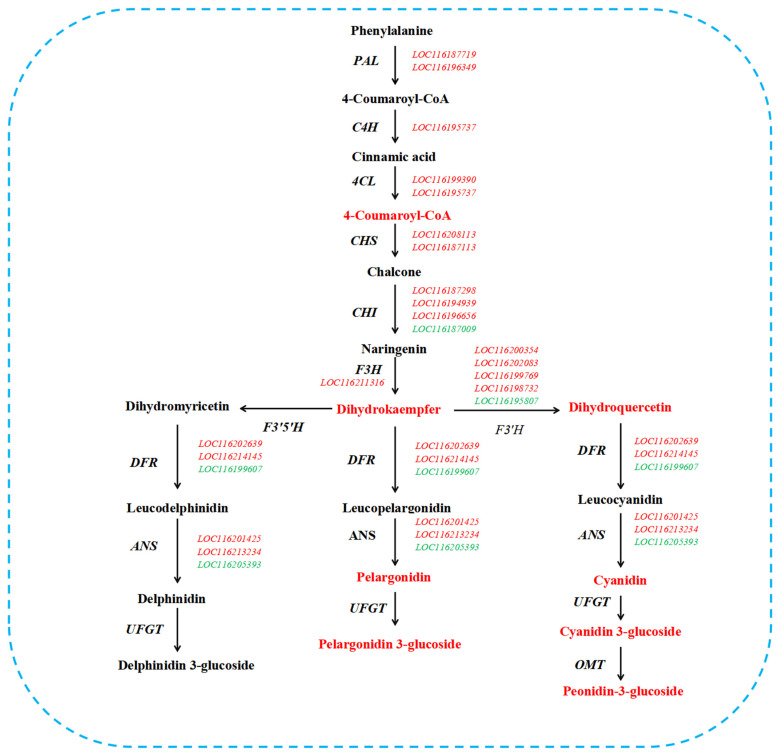
Expression patterns of the DEGs and DEMs involved in anthocyanin synthesis in Tunisia petals. Red represents upregulated expression of genes or metabolites, green represents downregulated expression of genes or metabolites.

The biosynthesis pathways of phenylpropane, flavonoids, and carotenoids are significantly enriched at both the transcriptional and metabolic levels (Figure 3B and Figure 7B). Therefore, we screened the key genes and metabolites that cause differences in pomegranate color from these three metabolic pathways. PAL, C4H, and 4CL are enzymes involved in the phenylpropane biosynthesis pathway, which is located upstream of the flavonoid biosynthesis pathway. The biosynthesis of anthocyanins requires the participation of the enzyme PAL, and studies on apples (*Malus pumila* Mill.) [31], tea trees (*Camellia sinensis*) [32], and carmine radish (*Raphanus sativus* L.) [33] have shown that the content of anthocyanins is positively correlated with the expression level of *PAL* genes. Transcriptome analysis of four varieties of *Impatiens uliginosa* flowers reveals that the *C4H* gene positively regulates the formation of red flowers [34]. The high expression of *PAL*, *C4H*, and *4CL* in purple peppers (*Capsicum annuum* L.) may be one of the reasons for the high content of anthocyanins [35,36]. In this study, two *PAL*, one *C4H*, and two *4CL* showed upregulated expression (Table 2), which may be the reason for the significant accumulation of 4-Coumaroyl-CoA in Tunisian petal (Table 3), ultimately leading to a significant increase in anthocyanins (Figure 9).

The anthocyanin biosynthesis pathway is a branch of the flavonoid synthesis pathway. The antisense *CHS* gene can interfere with the synthesis of anthocyanins in petunias (*Petunia hybrida*), resulting in a change in petunia petal color to white [37]. During the formation of red petals in hawthorn (*Crataegus pinnatifida*), the transcriptional level of *CHS* is significantly positively correlated with anthocyanin content [38]. In *Cymbidium orchid*, the expression level of *ChCHS* in the red variety is higher than that in other color varieties [39]. These results indicate that the *CHS* gene plays a crucial role in the coloring process of red petals, which is consistent with the upregulated expression of the two *CHS* genes in Tunisia petals observed in this study (Table 2). Inhibiting the expression of the *CHI* gene will increase the content of chalcones, block the flavonoid synthesis pathway, and cause the petal color to become lighter [40]. *CnCHI4* overexpression significantly increased flavonoid production in *Nicotiana tabacum* [41]. In the biosynthesis of anthocyanins in *Antirrhinum majus*, it has been found that the inhibition of *F3H* gene expression blocks the anthocyanin pathway, resulting in a change in flower color to white [42]. Inhibiting the expression of the *F3H* gene in carnation (*Dianthus caryophyllus*) will cause the flower color to fade or even turn white, and no anthocyanins were detected in the white-flowered plants [43]. In this study, one *F3H* gene and three *CHI* genes showed upregulated expression (Table 2), which may be the reason for the significant accumulation of dihydrokaempferol in Tunisian petals (Table 3), ultimately leading to a significant increase in anthocyanin (Figure 9).

Changes in the expression level of the *F3′H* gene can affect the variation in anthocyanin content in plants and the color status of the plant body. Using CRISPR/Cas9 to knock out the F3′H gene in a red-flowering poinsettia (*Euphorbia pulcherrima*) variety, the bract color of the transgenic plants changed from bright red to bright red/orange, and their anthocyanin levels were significantly reduced compared to the wild type [44]. The flowers of transgenic tobacco lines overexpressing the apple *F3′H* gene exhibit enhanced red pigment deposition. *DFR* is involved in the formation of colorless anthocyanins [45]. Functional validation of the separated *DFR* gene was conducted in model plants, and the transgenic plant color changed from white to red [46,47]. After the expression of the *DFR* gene is inhibited in *Camellia nitidissima* and *Allium cepa*, anthocyanin synthesis is blocked, and the colors of the flower and onion skin turn yellow [48,49]. The ANS enzyme converts colorless anthocyanins into colored anthocyanins, and inhibiting *ANS* gene expression can make the color lighter or whiter [50]. The formation of white flowers in Vanda hybrids may be related to the absence of the *ANS* gene [51]. Researchers measured the expression levels of *ANS*-encoding genes in mulberry (*Morus alba* L.) fruits of purple and white varieties and found that the *ANS* gene is only highly expressed in the flesh of purple mulberry fruits [52]. In this study, three *F3′H*, two *DFR*, and two *ANS* were upregulated, which may be an important reason for the high anthocyanin content and red color of Tunisian petals (Figure 9). In addition, metabolites in Tunisian petals, including pelargonidin, cyanidin, cyanidin 3-O-glucoside, peonidin 3-O-glucoside, and pelargonidin 3-O-glucoside, have high expression levels, which may be the reason for the red color of Tunisian petals (Figure 9).

Carotenoids give plants their yellow to red color and play a significant role in the diversity of flower color. PSY is the most important rate-limiting enzyme in the entire carotenoid metabolism pathway. After silencing *OgPSY* in the Oncidium orchid using RNA interference technology, the content of carotenoids in the petals decreased, and the petals changed from yellow to white [53]. During the ripening process of tomato (*Solanum lycopersicum*) fruits, the transcriptional level of *PSY1* significantly increases, leading to a substantial accumulation of lycopene [54]. However, when *PSY1* is mutated and inactivated or silenced by VIGS, the total content of carotenoids in tomatoes decreases significantly [55,56]. LCYE and LCYB are key branching enzymes that catalyze the cyclization reaction of lycopene, determining the content and composition of carotenoids in plant tissues [15]. After the loss of *LCYE* gene function in the *Arabidopsis thaliana lut2* mutant, the synthesis of β,β-branch carotenoids (violaxanthin, antheraxanthin, and zeaxanthin) significantly increased [57]. In this study, two *PSY* genes and one *LCYB* gene were upregulated in Tunisia petals, which is consistent with the high carotenoid content of Tunisia petals.

### 3.3. Key TFs Related to Pomegranate Petal Pigment

Many MYB transcription factors are involved in the positive regulation of anthocyanin biosynthesis [58]. In apples, overexpression of *MdMYB3* promotes upregulation of the *CHS*, *CHI*, *UFGT*, and *FLS* genes, and it was found that the expression of *MdMYB3* was higher in red-skinned apples than in yellow-skinned apples. In addition, tobacco plants converted to *MdMYB3* have darker flower colors than wild-type plants [59]. On the other hand, some MYB transcription factors can also negatively regulate the biosynthesis of anthocyanins [60]. The R2R3-MYB transcription factor *VvMYBC2L2* gene was found in grapes that can negatively regulate anthocyanin biosynthesis [61]. Transcription factors bHLH and MYB usually work together to activate the synthesis of anthocyanins [62]. When *Lc* (*bHLH*)/*C1* (*MYB*) was overexpressed individually in the petals of the Cymbidium hybrid “Jung Frau dos Pueblos”, only a few scarlet spots were produced [63]. However, when both factors were overexpressed simultaneously, hundreds of intense red spots were generated [63]. The structural genes involved in anthocyanin biosynthesis in plants are regulated by the MBW complex formed by the interaction of MYB, bHLH, and WD40. The MBW complex mainly regulates the biosynthesis of anthocyanins by modulating the transcriptional abundance of downstream structural genes involved in anthocyanin biosynthesis [64]. In blueberries, the expressions of *VcMYBL1*, *VcbHLH1*, and *VcWDL2* are positively correlated with the accumulation of anthocyanins and changes in color [65]. WRKY can regulate the anthocyanin synthesis pathway together with the MBW complex [66]. A study on the white and purple petals of two varieties of Orchids (*Phalaenopsis amabilis*) found that transcription factors such as PaWRKY, PaMADS, and PabZIP are all related to flower color formation [67]. The bZIP transcription factor MdHY5 in apples has been confirmed to promote anthocyanin biosynthesis by directly activating *MdMYB1/10* [68]. In this study, we identified a total of 18 differentially expressed transcription factor genes between Tunisia and White pomegranates, including *MYB*, *bZIP*, *MADS*, *WRKY*, *NAC*, and *bHLH*. One *MYB* gene was downregulated, but all the others were upregulated. We speculate that these differentially expressed transcription factors may be involved in the formation of pomegranate flower colors.

### 3.4. Effect of Caffeic Acid on Anthocyanin Content

Caffeic acid and its derivatives can interact with flavonoids such as anthocyanins to enhance the stability of petal color [69,70]. Research on blueberries has found that both caffeic acid and p-coumaric acid can enhance the color stability of blueberry anthocyanins [71]. PAL and C4H are two key enzymes involved in the biosynthesis of caffeic acid and its derivatives [72]. In this study, we observed upregulated expressions of the *PAL* and *C4H* genes at the transcriptional level. Additionally, in the metabolome analysis, we found that caffeic acid and ferulic acid (a derivative of caffeic acid) were also upregulated (Table S5). The results suggest that caffeic acid and its derivatives may also have an impact on the stability of anthocyanins, but further research is needed.

## 4. Materials and Methods

### 4.1. Plant Materials

This study used the “Tunisia” and “White” pomegranate varieties as materials, both of which were obtained from the Zhengzhou Fruit Research Institute, Chinese Academy of Agricultural Sciences, Zhengzhou, China. The experiment was conducted at Jiangsu Agri-animal Husbandry Vocational College (Taizhou, China). The two pomegranate varieties were planted and managed according to the local production practices in Taizhou, China. The blooming stage was defined as the period during which 50% of the pomegranate flowers were open [20]. For metabolomic and physiological studies, six replicates were set up for each pomegranate variety, with each replicate consisting of the petals from one pomegranate tree. For transcriptome studies, three replicates were set up for each pomegranate variety, with each replicate consisting of a uniform mixture of petals from two pomegranate trees. The petals from the blooming stage were frozen in liquid nitrogen and stored in an ultra-low-temperature (−80 °C) freezer for subsequent experiments.

### 4.2. Measurement of Physiological Indicators

#### 4.2.1. Measurement of Petal Color

The color index of petals was measured using a colorimeter (Konica Minolta CR-10 Plus, Konica Minolta, Shanghai, China), with specific operational steps following the instructions [73]. We obtained the L*, a*, and b* values of the two petal samples, and calculated the saturation C* according to the following formula: C* = (a^2^ + b^2^)^1/2^.

#### 4.2.2. Measurement of Anthocyanin, Flavonoid, and Carotenoid Content

The determination of anthocyanin content was conducted according to the method described by An et al. [74]. First, 1 g of fresh sample was ground into powder in liquid nitrogen and transferred to a 10 mL centrifuge tube. Then, 5 mL of 1% hydrochloric acid methanol solution (concentration expressed as a percentage by volume) was added, and the mixture was sealed with aluminum foil and stored in a refrigerator at 4 °C overnight. After centrifugation at 8000 rpm for 10 min, the supernatant was transferred to a cuvette, and the absorbance was measured at 530 nm, 620 nm, and 650 nm using a spectrophotometer. The anthocyanin content = ODλ ÷ ξλ × V ÷ m × 10^6^. In the formula, ODλ = (OD_530_ − OD_620_) − 0.1 × (OD_650_ − OD_620_); ξλ = 4.62 × 10^4^; V represents the volume of the extraction solution; and m denotes the mass of plant tissue.

Flavonoid content was measured using a flavonoid assay kit (Solarbio, BC1330, Beijing, China). We took 0.1 g of petal sample, strictly following the manufacturer’s guidelines, for pre-treatment, measurement, and flavonoid content calculation.

Carotenoid content was measured using a carotenoid assay kit (Solarbio, BC4330, Beijing, China). We took 0.1 g of petal sample, strictly following the manufacturer’s guidelines, for pre-treatment, measurement, and carotenoid content calculation.

#### 4.2.3. Measurement of Sucrose Content

Sucrose content was measured using a sucrose assay kit (Solarbio, BC2460, Beijing, China). We took 0.1 g of petal sample, strictly following the manufacturer’s guidelines, for pre-treatment, measurement, and sucrose content calculation.

### 4.3. Transcriptome Analysis

#### 4.3.1. Library Construction and Sequencing

Fresh petal samples of pomegranate were sent to Medway Metabolic Biotechnology Co., Ltd. (Wuhan, China) for transcriptome analysis. The total RNA was isolated and purified using the RNAprep Pure Plant Kit (Tiangen Biotech, Beijing, China), according to the manufacturer’s instructions. The RNA content of each sample was quantified using Qubit 4.0 (Thermo Fisher, Waltham, MA, USA). RNA integrity (RIN > 7.0) was determined using a Bioanalyzer 2100 (Agilent, Santa Clara, CA, USA). mRNA was enriched using magnetic beads with Oligo (dT), and this mRNA served as a template for reverse transcription synthesis of double-stranded cDNA. The double-stranded cDNA underwent purification, end repair, A-tail addition, and ligation of sequencing adapters, followed by PCR amplification to construct a cDNA library. After the cDNA library concentration and fragment size were tested to be qualified, sequencing was performed using the Illumina HiSeqTM 2000 platform (Illumina, San Diego, CA, USA).

#### 4.3.2. Bioinformatics Analysis

Clean reads were obtained by filtering the raw data using fastp (v0.23.2) software. The filtering criteria for clean reads were (1) removing reads with adapters; (2) removing reads with N content exceeding 10%; and (3) removing low-quality (Q ≤ 20) reads. All subsequent analyses are based on clean reads. We used Trinity (v2.15.1) software to assemble clean reads to obtain transcripts and then used Corset (v1.09) software to cluster and remove redundancy from the assembled transcripts. We aligned the UniGene sequence with KEGG, NR, SWISS-PROT, GO, KOG, and TrEMBL databases using DIAMOND software (v2.0.15) to obtain annotation information for UniGene [75]. The identification of differentially expressed genes was performed using DESeq2 (1.22.2), and the screening criteria for differentially expressed genes were |log2Fold Change| ≥ 1, with FDR < 0.05. Gene Ontology (GO) enrichment analysis and KEGG enrichment analysis of the DEGs were carried out with reference to Wang’s method [76]. Transcription factor prediction was conducted using iTAK (1.7a) software [77].

#### 4.3.3. qRT-PCR Analysis

To validate the RNA-Seq data analysis, the total RNA used for transcriptome sequencing was also used for qRT-PCR analysis for each biological replicate. We selected 10 DEGs from the RNA-Seq for qRT-PCR assays. Primers for qRT-PCR were designed using Primer 5.0 software (Table S4); the *PgActin* gene was used as an internal reference gene [78]. The *PgActin* primers were 5′-AGTCCTCTTCCAGCCATCTC-3′ (forward) and 3′CACTGAGCACAATGTTTCCA-5′ (reverse). The qRT-PCR conditions were set based on the following parameters: 10 min at 95 °C, 15 s at 95 °C (40 cycles of denaturation), 15 s for annealing at 60 °C, and 20 s for extension at 72 °C. The relative expression level of the genes for qRT-PCR was calculated according to the 2^−∆∆CT^ method. Three biological replicates (with three technical replicates for each biological replicate) were analyzed for each sample.

### 4.4. Metabolome Analysis

#### 4.4.1. Dry Sample Extraction

Using vacuum freeze-drying technology, we placed the petal samples in a lyophilizer (Scientz-100F, Scientz, Beijing, China), and then ground (30 Hz, 1.5 min) the samples to a powder form by using a grinder (MM 400, Retsch, Haan, Germany). Next, we weighed 50 mg of sample powder using an electronic balance (MS105DM) and added 1200 μL of −20 °C pre-cooled 70% methanolic aqueous internal standard extract. We vortexed once every 30 min for 30 s, for a total of 6 times. After centrifugation (rotation speed 12,000 rpm, 3 min), the supernatant was aspirated, and the sample was filtered through a microporous membrane (0.22 μm pore size) and stored in the injection vial for UPLC-MS/MS analysis.

#### 4.4.2. HPLC Conditions

All samples were subjected to two LC/MS methods. One aliquot was analyzed using positive ion conditions and was eluted from a T3 column (Waters ACQUITY Premier HSS T3 Column 1.8 µm, 2.1 mm × 100 mm) using 0.1% formic acid in water as solvent A and 0.1% formic acid in acetonitrile as solvent B in the following gradient: 5 to 20% in 2 min, increased to 60% in the following 3 min, increased to 99% in 1 min and held for 1.5 min, then returned to 5% mobile phase B within 0.1 min, held for 2.4 min. The analytical conditions were as follows: column temperature, 40 °C; flow rate, 0.4 mL/min; injection volume, 4 μL. Another aliquot was analyzed using negative ion conditions and was the same as the elution gradient of the positive mode.

#### 4.4.3. MS Conditions

The data acquisition was operated using the information-dependent acquisition (IDA) mode using Analyst TF 1.7.1 Software (SCIEX, Concord, ON, Canada). The source parameters were set as follows: ion source gas 1 (GAS1) was 50 psi; ion source gas 2 (GAS2) was 50 psi; curtain gas (CUR) was 25 psi; temperature (TEM) was 550 °C; declustering potential (DP) was 60 V or −60 V in the positive or negative mode, respectively; and ion spray voltage floating (ISVF) was 5000 V or −4000 V in the positive or negative mode, respectively. The TOF MS scan parameters were set as follows: mass range was 50–1000 Da; accumulation time was 200 ms; and dynamic background subtract was on. The product ion scan parameters were set as follows: mass range was 25–1000 Da; accumulation time was 40 ms; collision energy was 30 or −30 V in the positive or negative mode, respectively; collision energy spread was 15; resolution was UNIT; charge state was 1 to 1; intensity was 100 cps; exclude isotopes within 4 Da; mass tolerance was 50 ppm; and maximum number of candidate ions to monitor per cycle was 18.

#### 4.4.4. Metabolomics Data Processing

The raw metabolomic data were converted to mzXML format using ProteoWizard v. 3. Multivariate statistical analysis was performed on the normalized data matrix using principal component analysis (PCA) and partial least squares discriminant analysis (OPLS-DA) to reveal differences between samples and potential metabolic biomarkers.

#### 4.4.5. Selected Differential Metabolites

For two-group analysis, differential metabolites were determined with VIP (VIP > 1) and absolute Log2FC (|Log2FC| ≥ 1.0). VIP values were extracted from the OPLS-DA results, which also contain score plots and permutation plots, and were generated using the R package MetaboAnalystR. The data were log transform (log_2_) and mean centering before OPLS-DA. To avoid overfitting, a permutation test (200 permutations) was performed.

#### 4.4.6. KEGG Enrichment Analysis of Differential Metabolites

Identified metabolites were annotated using the KEGG Compound database (http://www.kegg.jp/kegg/compound/; accessed on 14 August 2024); annotated metabolites were then mapped to the KEGG Pathway database (http://www.kegg.jp/kegg/pathway.html; accessed on 25 August 2024).

### 4.5. Data Analysis

Data statistics and analysis were conducted using the software Excel 2019 (Microsoft Corporation, Redmond, WA, USA) and SPSS 25.0 (SPSS Corporation, New York, NY, USA), and Duncan’s method was employed to test the significance of differences.

## 5. Conclusions

In summary, we investigated the color difference mechanism between Tunisian pomegranate petals (red) and White pomegranate petals (white) by combining physiological, transcriptomic, and metabolomic analyses. The accumulation of anthocyanins and carotenoids is the primary reason for the red color of Tunisia petals. Caffeic acid and its derivatives may also play a role in the stability of Tunisia pomegranate petal color. The upregulation of genes in the anthocyanin biosynthesis pathway, such as *PAL*, *C4H*, *4CL*, *CHS*, *CHI*, *F3H*, *F3′H*, *DFR*, and *ANS*, is a crucial factor contributing to anthocyanin accumulation. The upregulated expression of genes in the carotenoid biosynthesis pathway, including *PSY* and *LCYB*, is a crucial factor in carotenoid accumulation. Transcription factor genes such as *MYB*, *bHLH*, *bZIP*, *MADS*, and *WRKY* may also have a positive regulatory effect on pigment accumulation. The red color of Tunisia’s petals is directly caused by changes in these genes. In the future, we will conduct functional validation on these key genes and further explore the mechanism of pomegranate color differences. These findings provide a molecular basis for future pomegranate color improvement and breeding.

## Figures and Tables

**Table 1:** Phenotypic parameters of pomegranate flower color.

Samples	Color	L*	a*	b*	C*
White	White	82.72 ± 0.53	−1.21 ± 0.15	8.16 ± 0.34	8.25 ± 0.23
Tunisia	Red	51.23 ± 0.45 ^#^	45.63 ± 0.88 ^#^	33.43 ± 1.27 ^#^	56.57 ± 0.89 ^#^

Note: # indicates significant differences at *p* < 0.05 level.

**Table 2:** DEGs related to pomegranate petal pigment.

Gene Name	Gene ID	Encoding Enzyme	Log2FC
*PAL1*	LOC116187719	phenylalanine ammonia-lyase [EC:4.3.1.24]	6.86
*PAL2*	LOC116196349	phenylalanine ammonia-lyase [EC:4.3.1.24]	4.37
*C4H*	LOC116195737	trans-cinnamate 4-monooxygenase [EC:1.14.14.91]	2.97
*4CL*	LOC116199390	4-coumarate--CoA ligase [EC:6.2.1.12]	2.69
*4CL*	LOC116195737	4-coumarate--CoA ligase [EC:6.2.1.12]	1.53
*CHS*	LOC116208113	chalcone synthase [EC:2.3.1.74]	7.60
*CHS*	LOC116187113	chalcone synthase [EC:2.3.1.74]	4.56
*CHI*	LOC116187298	chalcone isomerase [EC:5.5.1.6]	5.98
*CHI*	LOC116194939	chalcone isomerase [EC:5.5.1.6]	6.76
*CHI*	LOC116196656	chalcone isomerase [EC:5.5.1.6]	3.25
*CHI*	LOC116187009	chalcone isomerase [EC:5.5.1.6]	−1.58
*F3H*	LOC116211316	naringenin 3-dioxygenase [EC:1.14.11.9]	5.47
*F3′H*	LOC116200354	flavonoid 3′-monooxygenase [EC:1.14.14.82]	4.73
*F3′H*	LOC116202083	flavonoid 3′-monooxygenase [EC:1.14.14.82]	5.46
*F3′H*	LOC116199769	flavonoid 3′-monooxygenase [EC:1.14.14.82]	3.49
*F3′H*	LOC116198732	flavonoid 3′-monooxygenase [EC:1.14.14.82]	7.69
*F3′H*	LOC116195807	flavonoid 3′-monooxygenase [EC:1.14.14.82]	−1.65
*DFR*	LOC116202639	bifunctional dihydroflavonol 4-reductase/flavanone 4-reductase [EC:1.1.1.219 1.1.1.234]	2.36
*DFR*	LOC116214145	bifunctional dihydroflavonol 4-reductase/flavanone 4-reductase [EC:1.1.1.219 1.1.1.234]	5.12
*DFR*	LOC116199607	bifunctional dihydroflavonol 4-reductase/flavanone 4-reductase [EC:1.1.1.219 1.1.1.234]	−1.96
*ANS*	LOC116201425	anthocyanidin synthase [EC:1.14.20.4]	6.32
*ANS*	LOC116213234	anthocyanidin synthase [EC:1.14.20.4]	4.69
*ANS*	LOC116205393	anthocyanidin synthase [EC:1.14.20.4]	−1.77
*PSY*	LOC116205576	15-cis-phytoene synthase [EC:2.5.1.32]	3.63
*PSY*	LOC116199475	15-cis-phytoene synthase [EC:2.5.1.32]	4.78
*LCYB*	LOC116201477	lycopene beta-cyclase [EC:5.5.1.19]	2.19

**Table 3:** DEMs related to pomegranate petal pigment.

Index	Metabolite	VIP	log2(FC)	Entry
MEDL02688	4-Coumaroyl-CoA	1.59	6.56	C00223
MW0132689	Dihydrokaempferol	1.76	3.58	C00974
MW0130123	Dihydroquercetin	2.23	3.84	C01617
MEDL00398	Pelargonidin	1.35	8.53	C05904
MW0130498	Cyanidin	2.39	6.80	C05905
MW0165534	Cyanidin 3-glucoside	3.53	3.82	C08604
MW0145361	Peonidin 3-glucoside	2.49	4.83	C12141
MW0154969	Pelargonidin 3-glucoside	1.58	6.18	C12137
MW0139490	Prephytoene diphosphate	1.53	3.56	C03427
MW0114048	Phytoene	1.26	4.12	C05421
MW0114088	7,8-Dihydro-beta-carotene	3.46	2.86	C16291

## Data Availability

All data are presented in this article.

## Supplementary Materials

The following supporting information can be downloaded at https://www.mdpi.com/article/10.3390/plants14050652/s1: Table S1. Quality statistics of sequencing data for blue fescue samples; Table S2. Statistics on alignment efficiency of RNA-seq; Table S3. Differentially expressed transcription factor; Table S4. Primers for key genes in qRT-PCR; Table S5. DEMs related to caffeic acid; Figure S1. Sample correlation analysis chart; Figure S2. QC sample mass spectrometry detection TIC overlap map; Figure S3. CV value distribution map of the samples; Figure S4. Principal component analysis (PCA) of all samples.

## References

[1] Singh B., Singh J.P., Kaur A., Singh N. (2018). Phenolic compounds as beneficial phytochemicals in pomegranate (*Punica granatum* L.) peel: A review. Food Chem..

[2] Mphahlele R.R., Fawole O.A., Makunga N.P., Opara U.L. (2016). Effect of drying on the bioactive compounds, antioxidant, antibacterial and antityrosinase activities of pomegranate peel. BMC Complement. Altern. Med..

[3] Babu K.D. (2010). Floral biology of pomegranate (*Punica granatum* L.). Pomegranate.

[4] Wang Y., Chen L., Yang Q., Hu Z., Guo P., Xie Q., Chen G. (2022). New insight into the pigment composition and molecular mechanism of flower coloration in tulip (*Tulipa gesneriana* L.) cultivars with various petal colors. Plant Sci..

[5] Miller R., Owens S.J., Rørslett B. (2011). Plants and colour: Flowers and pollination. Opt. Laser Technol..

[6] Tanaka Y., Sasaki N., Ohmiya A. (2008). Biosynthesis of plant pigments: Anthocyanins, betalains and carotenoids. Plant J..

[7] Enaru B., Drețcanu G., Pop T.D., Stǎnilǎ A., Diaconeasa Z. (2021). Anthocyanins: Factors affecting their stability and degradation. Antioxidants.

[8] He F., Mu L., Yan G.-L., Liang N.-N., Pan Q.-H., Wang J., Reeves M.J., Duan C.-Q. (2010). Biosynthesis of anthocyanins and their regulation in colored grapes. Molecules.

[9] Jaakola L. (2013). New insights into the regulation of anthocyanin biosynthesis in fruits. Trends Plant Sci..

[10] Busche M., Pucker B., Weisshaar B., Stracke R. (2023). Three R2R3-MYB transcription factors from banana (*Musa acuminata*) activate structural anthocyanin biosynthesis genes as part of an MBW complex. BMC Res. Notes.

[11] Xu Y., Zheng H., Wang Q., Khalil-Ur-Rehman M., Meng L., Tao J. (2019). Comparison among ‘Benitaka’ grape, ABA-treated ‘Benitaka’, and its deeper-colored bud mutation revealed the coloring mechanisms on grapes. J. Plant Interact..

[12] Bhatt T., Patel K. (2020). Carotenoids: Potent to prevent diseases review. Nat. Prod. Bioprospecting.

[13] Sun T., Rao S., Zhou X., Li L. (2022). Plant carotenoids: Recent advances and future perspectives. Mol. Hortic..

[14] Sandmann G. (2021). Diversity and origin of carotenoid biosynthesis: Its history of coevolution towards plant photosynthesis. New Phytol..

[15] Sun T., Li L. (2020). Toward the ‘golden’ era: The status in uncovering the regulatory control of carotenoid accumulation in plants. Plant Sci..

[16] Ma X., Yu Y.-N., Jia J.-H., Li Q.-H., Gong Z.-H. (2022). The pepper MYB transcription factor CaMYB306 accelerates fruit coloration and negatively regulates cold resistance. Sci. Hortic..

[17] Liu R., Song J., Liu S., Chen C., Zhang S., Wang J., Xiao Y., Cao B., Lei J., Zhu Z. (2021). Genome-wide identification of the Capsicum bHLH transcription factor family: Discovery of a candidate regulator involved in the regulation of species-specific bioactive metabolites. BMC Plant Biol..

[18] Liang M.-H., Li X.-Y. (2023). Involvement of transcription factors and regulatory proteins in the regulation of carotenoid accumulation in plants and Algae. J. Agric. Food Chem..

[19] Bar-Ya’akov I., Tian L., Amir R., Holland D. (2019). Primary metabolites, anthocyanins, and hydrolyzable tannins in the pomegranate fruit. Front. Plant Sci..

[20] Luo X., Cao D., Li H., Zhao D., Xue H., Niu J., Chen L., Zhang F., Cao S. (2018). Complementary iTRAQ-based proteomic and RNA sequencing-based transcriptomic analyses reveal a complex network regulating pomegranate (*Punica granatum* L.) fruit peel colour. Sci. Rep..

[21] Wang X., Yang C., Zhu W., Weng Z., Li F., Teng Y., Zhou K., Qian M., Deng Q. (2024). Transcriptomic Analysis Reveals the Mechanism of Color Formation in the Peel of an Evergreen Pomegranate Cultivar ‘Danruo No. 1’ During Fruit Development. Plants.

[22] Zhao X., Yuan Z., Fang Y., Yin Y., Feng L. (2013). Characterization and evaluation of major anthocyanins in pomegranate (*Punica granatum* L.) peel of different cultivars and their development phases. Eur. Food Res. Technol..

[23] Li B., Lin J., Zheng Z., Duan H., Li D., Wu M. (2019). Effects of different drying methods on drying kinetics and physicochemical properties of *Chrysanthemum morifolium* Ramat. Int. J. Agric. Biol. Eng..

[24] Önder S., Tonguç M., Önder D., Erbaş S., Mutlucan M. (2023). Flower color and carbohydrate metabolism changes during the floral development of *Rosa damascena*. South Afr. J. Bot..

[25] Bento J.A.C., Ferreira K.C., de Oliveira A.L.M., Lião L.M., Caliari M., Júnior M.S.S. (2019). Extraction, characterization and technological properties of white garland-lily starch. Int. J. Biol. Macromol..

[26] Wang Y., Zhang C., Dong B., Fu J., Hu S., Zhao H. (2018). Carotenoid accumulation and its contribution to flower coloration of *Osmanthus fragrans*. Front. Plant Sci..

[27] Zhang D., Xie A., Yang X., Yang L., Shi Y., Dong L., Lei F., Sun L., Bao M., Sun X. (2023). Analysis of physiological and biochemical factors affecting flower color of herbaceous peony in different flowering periods. Horticulturae.

[28] Yamagishi M., Ihara H., Arakawa K., Toda S., Suzuki K. (2014). The origin of the *LhMYB12* gene, which regulates anthocyanin pigmentation of tepals, in Oriental and Asiatic hybrid lilies (*Lilium* spp.). Sci. Hortic..

[29] Télef N., Stammitti-Bert L., Mortain-Bertrand A., Maucourt M., Carde J.P., Rolin D., Gallusci P. (2006). Sucrose deficiency delays lycopene accumulation in tomato fruit pericarp discs. Plant Mol. Biol..

[30] Xia H., Zhu L., Zhao C., Li K., Shang C., Hou L., Wang M., Shi J., Fan S., Wang X. (2020). Comparative transcriptome analysis of anthocyanin synthesis in black and pink peanut. Plant Signal. Behav..

[31] Ju Z.-G., Yuan Y.-B., Liou C.-L., Xin S.-H. (1995). Relationships among phenylalanine ammonia-Iyase activity, simple phenol concentrations and anthocyanin accumulation in apple. Sci. Hortic..

[32] Chen X., Wang P., Gu M., Hou B., Zhang C., Zheng Y., Sun Y., Jin S., Ye N. (2022). Identification of PAL genes related to anthocyanin synthesis in tea plants and its correlation with anthocyanin content. Hortic. Plant J..

[33] Zhang Z., Sun C., Yao Y., Mao Z., Sun G., Dai Z. (2019). Red anthocyanins contents and the relationships with phenylalanine ammonia lyase (PAL) activity, soluble sugar and chlorophyll contents in carmine radish (*Raphanus sativus* L.). Hortic. Sci..

[34] Zhang X., Tan Y., Li X., Liu Z., Li F., Huang H., Huang M. (2024). Analysis of Transcriptome and Expression of *C4H* and *FLS* Genes on Four Flower Colors of *Impatiens uliginosa*. Horticulturae.

[35] Meng Y., Zhang H., Fan Y., Yan L. (2022). Anthocyanins accumulation analysis of correlated genes by metabolome and transcriptome in green and purple peppers (*Capsicum annuum*). BMC Plant Biol..

[36] Liu Y., Lv J., Liu Z., Wang J., Yang B., Chen W., Ou L., Dai X., Zhang Z., Zou X. (2020). Integrative analysis of metabolome and transcriptome reveals the mechanism of color formation in pepper fruit (*Capsicum annuum* L.). Food Chem..

[37] Van Der Krol A.R., Mur L.A., de Lange P., Mol J.N., Stuitje A.R. (1990). Inhibition of flower pigmentation by antisense *CHS* genes: Promoter and minimal sequence requirements for the antisense effect. Plant Mol. Biol..

[38] Tai D., Tian J., Zhang J., Song T., Yao Y. (2014). A Malus crabapple chalcone synthase gene, *McCHS*, regulates red petal color and flavonoid biosynthesis. PLoS ONE.

[39] Wang L., Albert N.W., Zhang H., Arathoon S., Boase M.R., Ngo H., Schwinn K.E., Davies K.M., Lewis D.H. (2014). Temporal and spatial regulation of anthocyanin biosynthesis provide diverse flower colour intensities and patterning in *Cymbidium orchid*. Planta.

[40] Nishihara M., Nakatsuka T., Yamamura S. (2005). Flavonoid components and flower color change in transgenic tobacco plants by suppression of chalcone isomerase gene. FEBS Lett..

[41] Yu S., Li J., Peng T., Ni S., Feng Y., Wang Q., Wang M., Chu X., Fan Z., Li X. (2022). Identification of chalcone isomerase family genes and roles of cnchi4 in flavonoid metabolism in *Camellia nitidissima*. Biomolecules.

[42] Martin C., Prescott A., Mackay S., Bartlett J., Vrijlandt E. (1991). Control of anthocyanin biosynthesis in flowers of *Antirrhinum majus*. Plant J..

[43] Zuker A., Tzfira T., Ben-Meir H., Ovadis M., Shklarman E., Itzhaki H., Forkmann G., Martens S., Neta-Sharir I., Weiss D. (2002). Modification of flower color and fragrance by antisense suppression of the flavanone 3-hydroxylase gene. Mol. Breed..

[44] Nitarska D., Boehm R., Debener T., Lucaciu R.C., Halbwirth H. (2021). First genome edited poinsettias: Targeted mutagenesis of flavonoid 3′-hydroxylase using CRISPR/Cas9 results in a colour shift. Plant Cell Tissue Organ.

[45] Han Y., Vimolmangkang S., Soria-Guerra R.E., Rosales-Mendoza S., Zheng D., Lygin A.V., Korban S.S. (2010). Ectopic expression of apple *F3′H* genes contributes to anthocyanin accumulation in the Arabidopsis *tt7* mutant grown under nitrogen stress. Plant Physiol..

[46] Li H., Qiu J., Chen F., Lv X., Fu C., Zhao D., Hua X., Zhao Q. (2012). Molecular characterization and expression analysis of *dihydroflavonol 4-reductase* (*DFR*) gene in *Saussurea medusa*. Mol. Biol. Rep..

[47] Polashock J.J., Griesbach R.J., Sullivan R.F., Vorsa N. (2002). Cloning of a cDNA encoding the cranberry dihydroflavonol-4-reductase (DFR) and expression in transgenic tobacco. Plant Sci..

[48] Kim S., Yoo K.S., Pike L.M. (2005). Development of a PCR-based marker utilizing a deletion mutation in the *dihydroflavonol 4-reductase* (*DFR*) gene responsible for the lack of anthocyanin production in yellow onions (*Allium cepa*). Theor. Appl. Genet..

[49] Zhou X.-W., Fan Z.-Q., Chen Y., Zhu Y.-L., Li J.-Y., Yin H.-F. (2013). Functional analyses of a flavonol synthase–like gene from *Camellia nitidissima* reveal its roles in flavonoid metabolism during floral pigmentation. J. Biosci..

[50] Nakamura N., Fukuchi-Mizutani M., Miyazaki K., Suzuki K., Tanaka Y. (2006). RNAi suppression of the anthocyanidin synthase gene in *Torenia hybrida* yields white flowers with higher frequency and better stability than antisense and sense suppression. Plant Biotechnol..

[51] Junka N., Kanlayanarat S., Buanong M., Wongchaochant S., Wongs-Aree C. (2011). Analysis of Anthocyanins and the Expression Patterns of Genes Involved in Biosynthesis in two Vanda Hybrids. Int. J. Agric. Biol..

[52] Li J., Lü R.-H., Zhao A.-C., Wang X.-L., Liu C.-Y., Zhang Q.-Y., Wang X.-H., Umuhoza D., Jin X.-Y., Lu C. (2014). Isolation and expression analysis of anthocyanin biosynthetic genes in *Morus alba* L. Biol. Plant..

[53] Liu Y.C., Yeh C.W., Chung J.D., Tsai C.Y., Chiou C.Y., Yeh K.W. (2019). Petal-specific RNAi-mediated silencing of the phytoene synthase gene reduces xanthophyll levels to generate new *Oncidium* orchid varieties with white-colour blooms. Plant Biotechnol. J..

[54] Ronen G., Cohen M., Zamir D., Hirschberg J. (1999). Regulation of carotenoid biosynthesis during tomato fruit development: Expression of the gene for lycopene epsilon-cyclase is down-regulated during ripening and is elevated in the mutant Delta. Plant J..

[55] Gady A.L., Vriezen W.H., Van de Wal M.H., Huang P., Bovy A.G., Visser R.G., Bachem C.W. (2012). Induced point mutations in the phytoene synthase 1 gene cause differences in carotenoid content during tomato fruit ripening. Mol. Breed..

[56] Fantini E., Falcone G., Frusciante S., Giliberto L., Giuliano G. (2013). Dissection of tomato lycopene biosynthesis through virus-induced gene silencing. Plant Physiol..

[57] Pogson B., McDonald K.A., Truong M., Britton G., DellaPenna D. (1996). Arabidopsis carotenoid mutants demonstrate that lutein is not essential for photosynthesis in higher plants. Plant Cell.

[58] Zhao A., Cui Z., Li T., Pei H., Sheng Y., Li X., Zhao Y., Zhou Y., Huang W., Song X. (2019). mRNA and miRNA expression analysis reveal the regulation for flower spot patterning in *Phalaenopsis* ‘Panda’. Int. J. Mol. Sci..

[59] Vimolmangkang S., Han Y., Wei G., Korban S.S. (2013). An apple MYB transcription factor, MdMYB3, is involved in regulation of anthocyanin biosynthesis and flower development. BMC Plant Biol..

[60] Fu Z., Wang L., Shang H., Dong X., Jiang H., Zhang J., Wang H., Li Y., Yuan X., Meng S. (2019). An R3-MYB gene of Phalaenopsis, *MYBx1*, represses anthocyanin accumulation. Plant Growth Regul..

[61] Zhu Z., Li G., Liu L., Zhang Q., Han Z., Chen X., Li B. (2018). A R2R3-MYB transcription factor, VvMYBC2L2, functions as a transcriptional repressor of anthocyanin biosynthesis in grapevine (*Vitis vinifera* L.). Molecules.

[62] Nakatsuka T., Suzuki T., Harada K., Kobayashi Y., Dohra H., Ohno H. (2019). Floral organ-and temperature-dependent regulation of anthocyanin biosynthesis in *Cymbidium hybrid* flowers. Plant Sci..

[63] Albert N.W., Arathoon S., Collette V.E., Schwinn K.E., Jameson P.E., Lewis D.H., Zhang H., Davies K.M. (2010). Activation of anthocyanin synthesis in *Cymbidium* orchids: Variability between known regulators. Plant Cell Tissue Organ.

[64] Xu W., Dubos C., Lepiniec L. (2015). Transcriptional control of flavonoid biosynthesis by MYB–bHLH–WDR complexes. Trends Plant Sci..

[65] Zhao M., Li J., Zhu L., Chang P., Li L., Zhang L. (2019). Identification and characterization of MYB-bHLH-WD40 regulatory complex members controlling anthocyanidin biosynthesis in blueberry fruits development. Genes.

[66] Lloyd A., Brockman A., Aguirre L., Campbell A., Bean A., Cantero A., Gonzalez A. (2017). Advances in the MYB–bHLH–WD repeat (MBW) pigment regulatory model: Addition of a WRKY factor and co-option of an anthocyanin MYB for betalain regulation. Plant Cell Physiol..

[67] Meng X., Li G., Gu L., Sun Y., Li Z., Liu J., Wu X., Dong T., Zhu M. (2020). Comparative metabolomic and transcriptome analysis reveal distinct flavonoid biosynthesis regulation between petals of white and purple *Phalaenopsis amabilis*. J. Plant Growth Regul..

[68] An J.-P., Qu F.-J., Yao J.-F., Wang X.-N., You C.-X., Wang X.-F., Hao Y.-J. (2017). The bZIP transcription factor MdHY5 regulates anthocyanin accumulation and nitrate assimilation in apple. Hortic. Res..

[69] Darias-Martín J., Martín-Luis B., Carrillo-López M., Lamuela-Raventós R., Díaz-Romero C., Boulton R. (2002). Effect of caffeic acid on the color of red wine. J. Agr. Food Chem..

[70] Qian B.-J., Liu J.-H., Zhao S.-J., Cai J.-X., Jing P. (2017). The effects of gallic/ferulic/caffeic acids on colour intensification and anthocyanin stability. Food Chem..

[71] Liu J., Zhuang Y., Hu Y., Xue S., Li H., Chen L., Fei P. (2020). Improving the color stability and antioxidation activity of blueberry anthocyanins by enzymatic acylation with p-coumaric acid and caffeic acid. LWT Food Sci. Technol..

[72] Berner M., Krug D., Bihlmaier C., Vente A., Müller R., Bechthold A. (2006). Genes and enzymes involved in caffeic acid biosynthesis in the actinomycete *Saccharothrix espanaensis*. J. Bacteriol..

[73] Zhou Y., Yin M., Abbas F., Sun Y., Gao T., Yan F., Li X., Yu Y., Yue Y., Yu R. (2022). Classification and association analysis of Gerbera (*Gerbera hybrida*) flower color traits. Front. Plant Sci..

[74] An X.-H., Tian Y., Chen K.-Q., Liu X.-J., Liu D.-D., Xie X.-B., Cheng C.-G., Cong P.-H., Hao Y.-J. (2015). *MdMYB9* and *MdMYB11* are involved in the regulation of the JA-induced biosynthesis of anthocyanin and proanthocyanidin in apples. Plant Cell Physiol..

[75] Zang H., Guo S., Dong S., Song Y., Li K., Fan X., Qiu J., Zheng Y., Jiang H., Wu Y. (2024). Construction of a Full-Length Transcriptome of Western Honeybee Midgut Tissue and Improved Genome Annotation. Genes.

[76] Wang A., Kang L., Yang G., Li Z. (2022). Transcriptomic and iTRAQ-Based Quantitative Proteomic Analyses of inap CMS in *Brassica napus* L. Plants.

[77] Zheng Y., Jiao C., Sun H., Rosli H.G., Pombo M.A., Zhang P., Banf M., Dai X., Martin G.B., Giovannoni J.J. (2016). iTAK: A program for genome-wide prediction and classification of plant transcription factors, transcriptional regulators, and protein kinases. Mol. Plant.

[78] Ono N.N., Bandaranayake P.C., Tian L. (2012). Establishment of pomegranate (*Punica granatum*) hairy root cultures for genetic interrogation of the hydrolyzable tannin biosynthetic pathway. Planta.

