# Supplementary material for: Transcriptome and Metabolome Analyses Reveal the Mechanism of Color Differences in Pomegranate (Punica granatum L.) Red and White Petals
Source: Plants (Basel). 2025 Feb 20;14(5):652. doi: 10.3390/plants14050652 (PMC11901741; doi:10.3390/plants14050652)
Supplement: Supplementary file 1 [file plants-14-00652-s001.zip › plants-3489521-Supplement of experimental data.pdf]

Table S1. Quality statistics of sequencing data for blue fescue samples

Sample	Raw Reads	Clean Reads	Clean Base(G)	Error Rate(%)	Q20(%)	Q30(%)	GC Content(%)
White-1	62982832	61036826	9.16	0.01	98.78	96.13	51.69
White-2	65286540	63736056	9.56	0.01	98.84	96.37	51.73
White-3	62173504	60829522	9.12	0.01	98.83	96.34	51.7
Tunisia-1	71690774	69564844	10.43	0.01	98.86	96.39	51.03
Tunisia-2	61125606	59937934	8.99	0.01	98.82	96.28	51.65
Tunisia-3	60161624	58388768	8.76	0.01	98.79	96.16	50.62

Table S2. Statistics on alignment efficiency of RNA-seq

Sample Name	Total Reads	Reads mapped	Uniq mapped reads	Multiple mapped reads
White-1	61036826	59839468(98.04%)	57229700(93.76%)	2609768(4.28%)
White-2	63736056	62450415(97.98%)	59672415(93.62%)	2778000(4.36%)
White-3	60829522	59670693(98.09%)	57214698(94.06%)	2455995(4.04%)
Tunisia-1	69564844	68256078(98.12%)	65089887(93.57%)	3166191(4.55%)
Tunisia-2	59937934	58775590(98.06%)	56508567(94.28%)	2267023(3.78%)
Tunisia-3	58388768	56745619(97.19%)	54691377(93.67%)	2054242(3.52%)

Table S3. Differentially expressed transcription factor

Transcription factor	Gene ID	Log2FC	Expression pattern
MYB	LOC116192039	6.57	up-regulated expression
	LOC116192184	4.56	up-regulated expression
	LOC116192393	5.34	up-regulated expression
	LOC116192630	1.85	up-regulated expression
	LOC116192926	3.42	up-regulated expression
	LOC116188020	-1.79	down-regulated expression
bHLH	LOC116187405	4.51	up-regulated expression
	LOC116187405	2.58	up-regulated expression
	LOC116188346	3.46	up-regulated expression
	LOC116188346	2.93	up-regulated expression
bZIP	LOC116192892	1.58	up-regulated expression
	LOC116192892	3.17	up-regulated expression
	LOC116194290	2.88	up-regulated expression
NAC	LOC116189180	3.18	up-regulated expression
	LOC116194533	5.33	up-regulated expression

MADS	LOC116190093	1.53	up-regulated expression
	LOC116194538	5.68	up-regulated expression
WRKY	LOC116192039	3.61	up-regulated expression

Table S4. Primers for key genes in qRT-PCR

Gens ID		Primer
PgPAL1-F	LOC116187719	GATGCAACCGTCAACACC
PgPAL1-R		ATGACCGATGATTGCCTT
Pg4CL-F	LOC116199390	GCCAAAGCCTCCAACACC
Pg4CL-R		ACTTCTGATGCCTCGGTT
PgC4H-F	LOC116195737	CCTCCATGATGCGAAGCTC
PgC4H-R		GCTTCGACGTCCAACACC
PgCHS-F	LOC116208113	CGTCTTCTGCACTACCTCC
PgCHS-R		TGGTACATCATGAGACGCTT
PgCHI-F	LOC116187009	CCCACCTTAATCCCGTCCA
PgCHI-R		TCCCTTTTCGTTCCGGCTTG
PgF3H-F	LOC116211316	CACAGCCCGACCTCACC
PgF3H-R		CCTCCATCACCGGCTTC
PgF3'H-F	LOC116195807	GGCCGCTCATGCACCT
PgF3'H-R		CCTCAACATCCGCCACCT
PgDFR-F	LOC116214145	CCGGCATCGCAAAGCTC
PgDFR-R		CACAGCCCCGACGAAC
PgANS-F	LOC116201425	CTCGGCCCTCTCGCTTG
PgANS-R		ACAGAACACCGCCCACGA
PgMYB-F	LOC116192039	TACCTTGCCAACTCCGTCT
PgMYB-R		CACATCATCGAATCGCCAGT

Table S5. DEMs related to caffeic acid

Index	Metabolite	VIP	log2(FC)	Entry
MW0139304	Ferulic acid	3.59	1.79	C01494
MW0105678	Caffeic acid	4.52	5.63	C01197

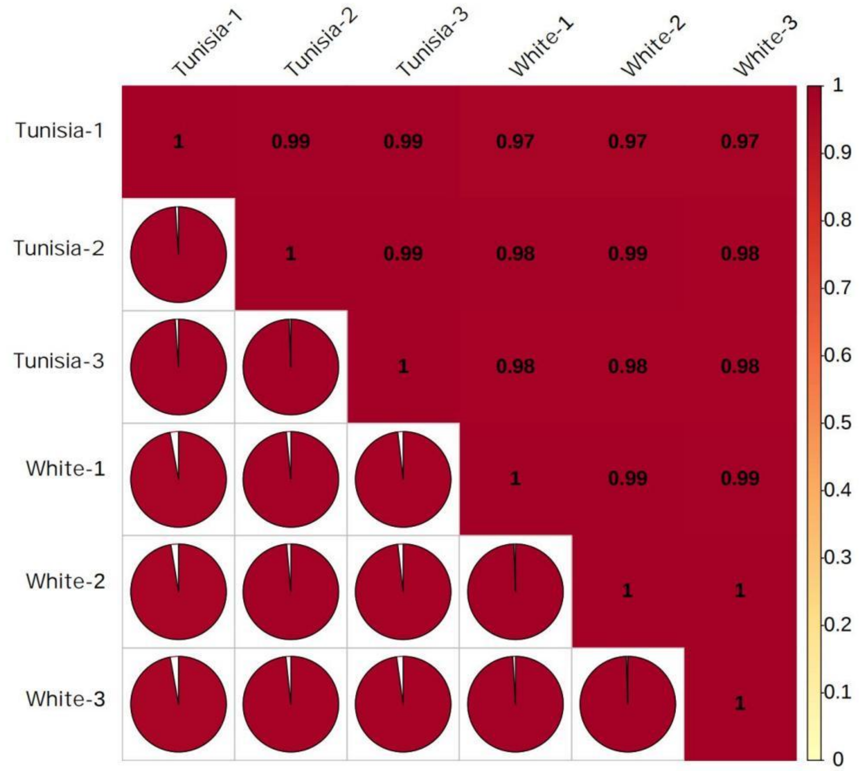

Figure S1. Sample correlation analysis chart

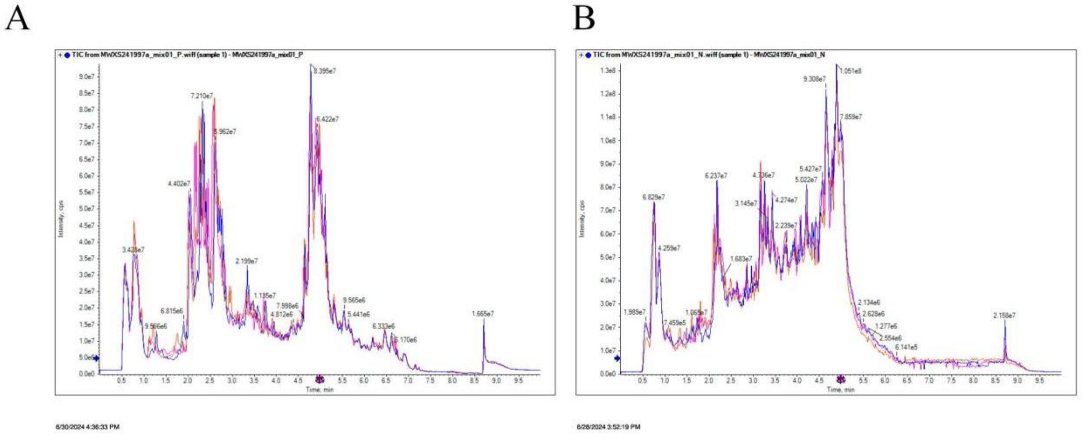

Figure S2. QC sample mass spectrometry detection TIC overlap map. A, Positive ion mode; B, Negative ion mode.

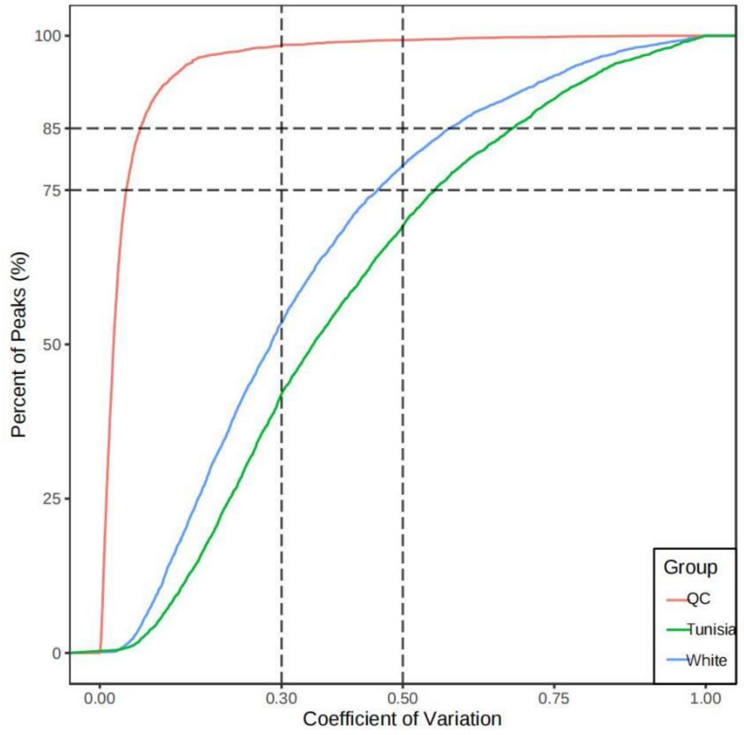

Figure S3. CV value distribution map of the samples

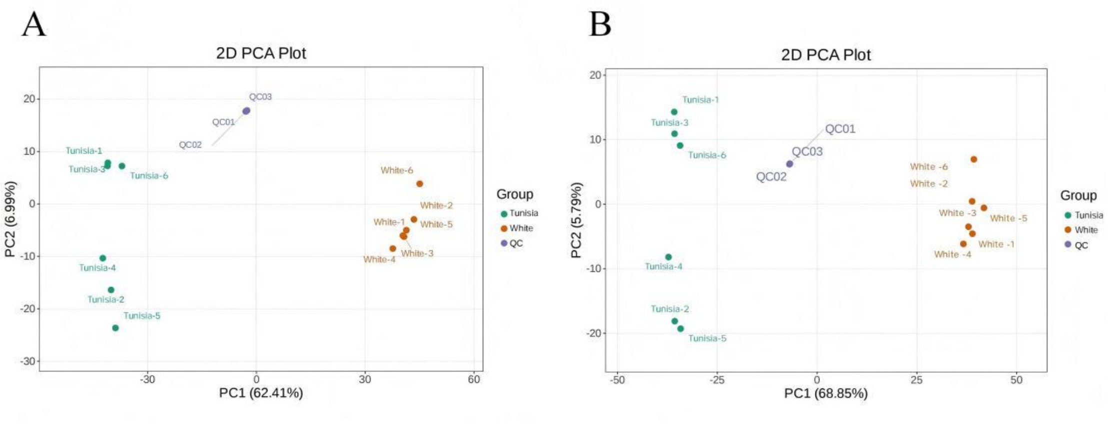

Figure S4. Principal component analysis (PCA) of all samples. A, Positive ion mode; B, Negative ion mode.
